# Lactoferrin Displays Stimulating and Protective Effects on Newly Isolated Phage vB_Sau-E: A New Perspective for Treatment of Staphylococcal Skin Infections

**DOI:** 10.3390/ph19060808

**Published:** 2026-05-22

**Authors:** Urszula Leszczyńska, Małgorzata Stasiłojć, Milena Grzenkowicz, Magdalena Narajczyk, Agnieszka Necel, Lidia Piechowicz, Katarzyna Kosznik-Kwaśnicka, Anna Żywicka

**Affiliations:** 1 Student Scientific Circle of Medical Microbiology, Department of Medical Microbiology, Faculty of Medicine, Medical University of Gdańsk, 80-204 Gdańsk, Poland; urszula.leszczynska@gumed.edu.pl; 2 Department of Cell Biology and Immunology, Intercollegiate Faculty of Biotechnology, University of Gdańsk and Medical University of Gdańsk, 80-211 Gdańsk, Poland; mstasilojc@gumed.edu.pl; 3 Department of Medical Microbiology, Faculty of Medicine, Medical University of Gdańsk, 80-204 Gdańsk, Poland; milenagrzenkowicz@gmail.com (M.G.); agnieszka.necel@gumed.edu.pl (A.N.); lidia.piechowicz@gumed.edu.pl (L.P.); 4 J.S. Hamilton Poland Sp. z o.o., 81-571 Gdynia, Poland; 5 Bioimaging Laboratory, Faculty of Biology, University of Gdańsk, 80-308 Gdańsk, Poland; magdalena.narajczyk@ug.edu.pl; 6 Department of Microbiology and Biotechnology, Faculty of Biotechnology and Animal Husbandry, West Pomeranian University of Technology in Szczecin, 71-270 Szczecin, Poland

**Keywords:** bacteriophages, skin infections, *Staphylococcus aureus*, MRSA, phage therapy

## Abstract

**Background/Objectives:** Skin and soft tissue infections (SSTIs) represent a significant clinical challenge, largely due to the high prevalence of antibiotic-resistant *Staphylococcus aureus*, particularly methicillin-resistant *S. aureus* (MRSA). Treatment is further complicated by biofilm formation, which reduces antibiotic efficacy. The limitations of conventional therapies highlight the need for alternative approaches. Phage therapy has emerged as a promising biological strategy; however, its effectiveness may be constrained by factors such as phage instability and biofilm regrowth. This study aimed to enhance phage-based treatment by combining a newly isolated phage, vB_Sau-E, with lactoferrin (Lf), a multifunctional protein of the innate immune system. **Methods:** Phage vB_Sau-E was characterized in terms of its infection dynamics and lytic activity. Biocompatibility was further examined using human skin cell lines. The potential effect of Lf was assessed by evaluating its impact on phage infectivity and stability under a range of environmental conditions and by checkerboard assay. **Results:** Phage vB_Sau-E belongs to the *Silviavirus* genus in the *Herelleviridae* family. It was shown to infect 12 out of 22 tested clinical MRSA isolates, with 10 strains identified as good hosts. The phage has a ~30 min life cycle, and ~50 progeny virions are released after bacterial cell lysis. We have also observed that Lf increased plating efficiency and enhanced phage stability at a pH of 5.5 and at −20° C. It also proved to have an additive antibacterial effect, though this was observed to be strain-dependent. **Conclusions:** Lactoferrin functions as a stabilizing adjuvant for phage vB_Sau-E. Its additive effect supports the development of more effective, biofilm-targeting therapies for staphylococcal SSTIs.

## 1. Introduction

The most common pathogen responsible for skin and soft tissue infections (SSTIs) is *Staphylococcus aureus* [1,2,3]. These infections range from mild, superficial lesions with localized manifestations to severe conditions such as necrotizing fasciitis or sepsis, which may lead to serious complications or death [4,5]. Methicillin-resistant *S. aureus* (MRSA) represents a major phenotype associated with both community-acquired and hospital-acquired infections, posing significant challenges to current treatment strategies and contributing to therapeutic failures [6,7]. Additionally, biofilm formation enables *Staphylococcus aureus* to persist in a protected, metabolically altered state until favorable conditions arise, at which point localized or systemic symptoms may develop. This environment also reduces susceptibility to antimicrobial agents and host immune responses, contributing to treatment failure [8].

Given the increasing occurrence of infections caused by antibiotic-resistant *S. aureus*, there is a growing need to explore alternative therapeutic approaches, such as phage therapy [9]. Bacteriophages are viruses that specifically infect and lyse bacteria. In contrast to conventional antibiotics, phages exhibit high host specificity, allowing them to target pathogenic bacteria without disrupting the host’s commensal microbiota [9]. Another reason in favor of using phages is that bacteria gain resistance to them approximately 10 times slower compared to antibiotics [10]. Phage therapy is also considered relatively safe, as bacteriophages are naturally present in the human body and are not typically associated with adverse inflammatory responses [10,11]. Finally, what is crucial for treating skin infections, phages are capable of biofilm eradication, e.g., due to the presence of polysaccharide depolymerase enzymes that enable access to the bacteria locked in the biofilm [9].

Recent clinical studies have demonstrated promising outcomes of phage therapy in the treatment of *S. aureus* infections, including those originating from bloodstream and soft tissue infections, with particularly high susceptibility observed among MRSA strains [12]. Unfortunately, the use of phages faces certain limitations, including a narrow spectrum of activity, the potential risk of toxins or antibiotic resistance gene transfer, and, more importantly, the re-growth of biofilm observed after treatment [13,14]. Considering foregoing restrictions, it seems promising to explore combinations of phages with other antimicrobial agents such as bacteriocins or nanoparticles to improve phage therapeutic outcomes [9,15].

Lactoferrin (Lf) is an iron-binding glycoprotein that constitutes an important component of the mammalian innate immune system. It is present in various bodily fluids, including blood, mucosal secretions, sweat, tears, and milk. Lactoferrin exhibits antimicrobial activity through mechanisms such as iron sequestration and disruption of microbial membranes, and it also possesses anti-biofilm properties, with preliminary research showing promising results when used in combination with bacteriophages [16,17]. Furthermore, as a natural component of the human immune system with documented immunomodulatory and wound-healing effects, Lf represents a biologically compatible therapeutic adjunct [18].

In this study, we characterize a newly isolated staphylococcal phage, vB_Sau-E, in terms of its host range, stability, and safety to human cells. Additionally, we investigated the combined application of lactoferrin with vB_Sau-E as a potential strategy for the treatment of staphylococcal skin infections. Our experimental data indicate that Lf enhances phage plating efficiency and improves phage stability under varying environmental conditions, including fluctuations in pH and temperature. In addition, an additive effect was observed in a strain-dependent manner among MRSA isolates. Nevertheless, further studies are required to evaluate the efficacy of this combination in more complex in vivo systems, including animal models and clinical trials.

## 2. Results

### 2.1. Phage vB_Sau-E Morphology

**Figure 1:**
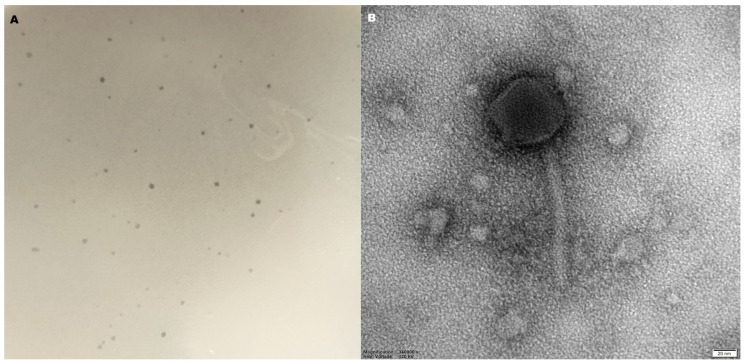
Plaque (**A**) and virion (**B**) morphology of phage vB_Sau-E.

Phage vB_Sau-E creates small, clear plaques on bacterial lawn, approx. 1 mm wide (Figure 1A). A TEM analysis revealed the phage to have a myovirus morphotype, with an icosahedral head (0.06 µm × 0.06 µm) and a long tail of ~0.15 µm in length (Figure 1B).

### 2.2. Genome and Phylogenetic Analysis

**Figure 2:**
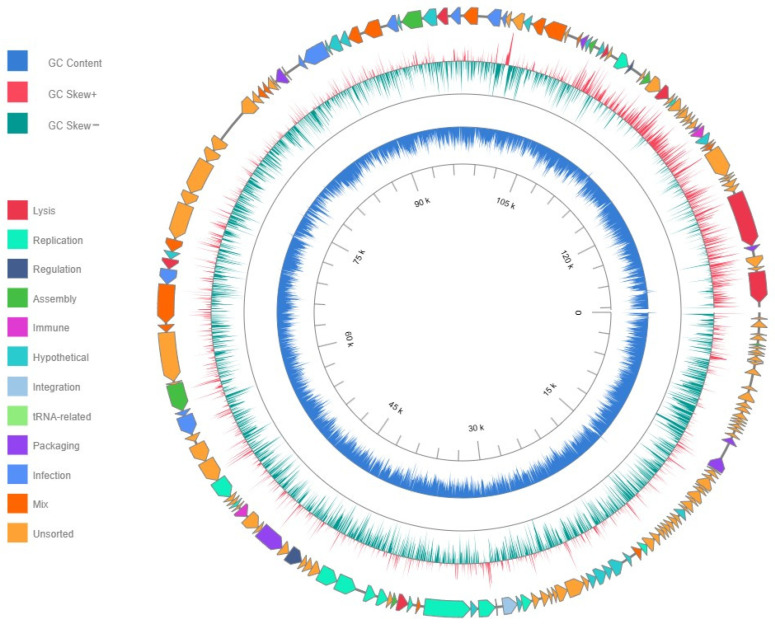
A map of the vB_Sau-E genome created by the PhageScope platform. The outer circle describes the potential functionality of hypothetical products. The inner colored circles show the GC parameters.

The analysis of the vB_Sau-E phage genome showed that it contains 129,312 bp with 30.1% of GC pairs. Functional predictions by the PhageScope platform revealed the presence of 171 open reading frames (ORFs), for which 96 possible functions were predicted (Figure 2). Most of the products (51) were identified as engaged in processes of infection, assembly, and replication. These include virion structural proteins, tail fiber proteins, and family A DNA polymerase. The analysis also indicated the production of eight products contributing to the lysis process, including endolysins with a LysM domain or amidase activity and enzymes from the glycosyl hydrolase family. Furthermore, host prediction pointed to *Staphylococcus aureus* as a host for the isolated phage, and its features indicate the lytic lifestyle. Additionally, it did not show the presence of genes of virulent factors or antimicrobial resistance.

**Figure 3:**
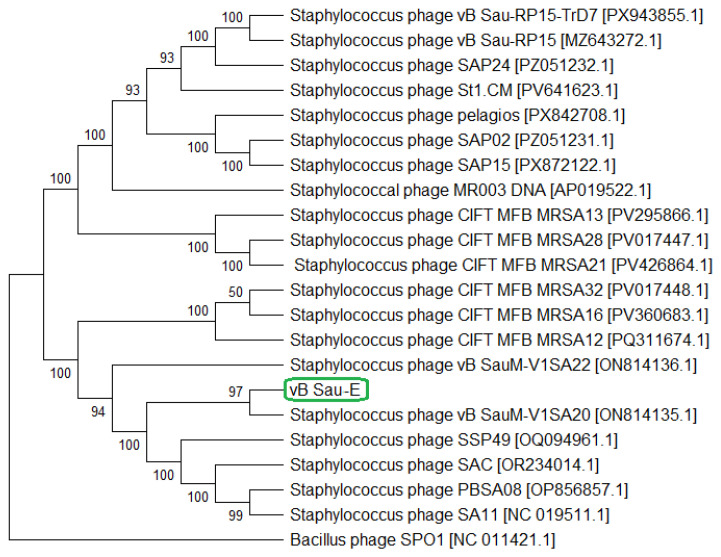
Phylogenetic relationships based on the complete genome sequence of phage vB_Sau-E. Multiple sequence alignment was performed using the MAFFT algorithm, and the phylogenetic tree was constructed in MEGA12 using the maximum-likelihood method with 1000 bootstrap replicates. Bootstrap support values are indicated at the corresponding nodes.

Phylogenetic analysis revealed that phage vB_Sau-E displays the closest genetic relation with the *Staphylococcus* phage vB_SauM-V1SA20 [ON814135.1] (coverage: 93%; identity, 97.32%). Thus, its similarity classifies the newly isolated phage into the *Silviavirus* genus, within the *Twortvirinae* subfamily, *Herelleviridae* family, and the *Caudoviricetes* class (Figure 3).

### 2.3. Host Range

A total of 22 MRSA strains isolated from skin or wound tissue were used to determine the host range and efficacy of plating (EOP) of vB_Sau-E phage. MRSA strain no. 70 was a primary host, and therefore, it has been used as a reference strain to calculate the EOP. It was observed that the phage was able to infect 12 out of 22 tested strains, with the majority of them being classified as a good host (with an average EOP value of ~20–25%) (Table 1). Only two strains, MRSA 366 and 199, were classified as poor hosts, with EOP of 6.93 ± 3.62% and EOP = 0.35 ± 0.09%, respectively (Table 1).

### 2.4. Phage vB_Sau-E Stability in Different Conditions

Across the tested temperature range, phage vB_Sau-E retained substantial viability (≥100% relative viability) at 25–37 °C. A moderate reduction was observed at 4 °C and 60 °C, whereas exposure to 95 °C resulted in complete loss of infectivity. Interestingly, the phage remained active, though at low titer (0.61% compared with the control) at −20 °C (Table 2). The phage was additionally inactivated at highly acidic and alkaline pHs, while incubation at a pH of 5.5 or 9 resulted in ≥50% loss of viability (Table 2). The stability of phage vB_Sau-E at different conditions is presented in Table 2.

### 2.5. Phage vB_Sau-E Life Cycle Characteristics

**Figure 4:**
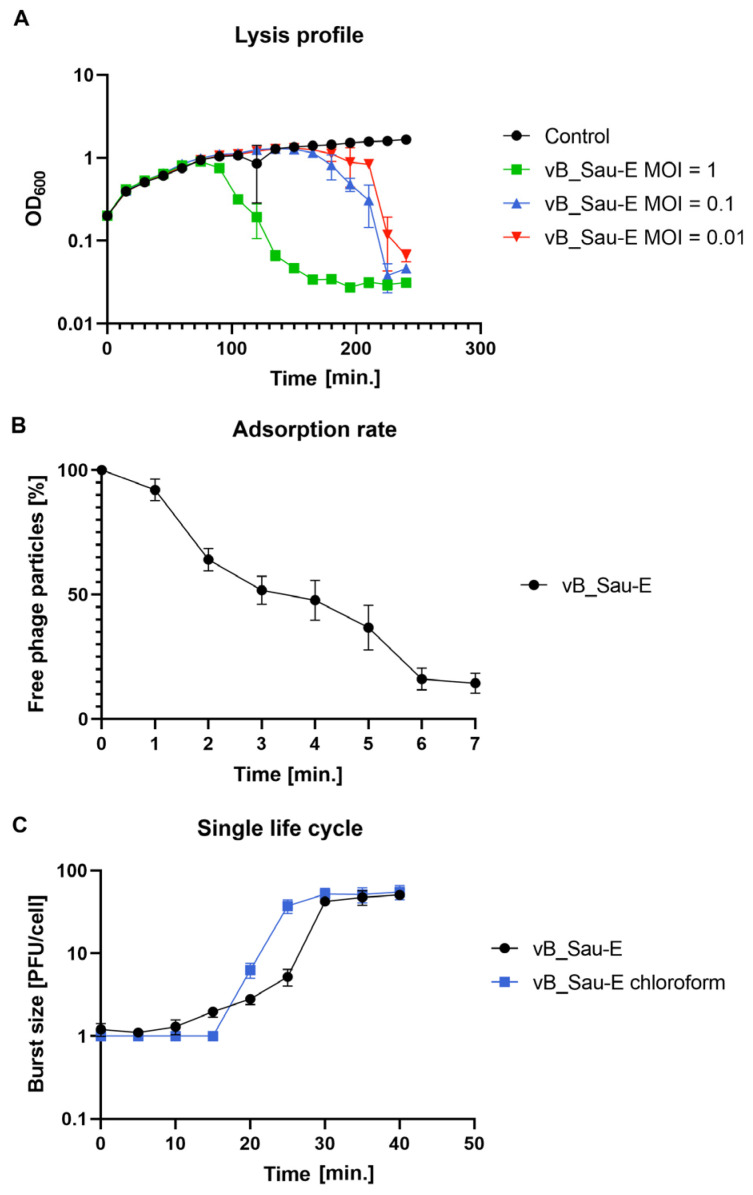
Life cycle characteristics of phage vB_Sau-E with MRSA 70 used as a host: lysis profile (**A**), adsorption rate (**B**) and life cycle kinetics (**C**). Arithmetic mean of triplicates with error bars representing SD.

The phage was shown to effectively lyse bacterial culture even at low MOI, although it took ~160 min for the phage lytic activity to be visible (as a decrease in optical density) at multiplicity of infection (MOI) of 0.01 and 0.1. In case of MOI = 1, the infected bacterial culture would lyse after ~90 min post-infection, and OD_600_ would stabilize after ~180 min (Figure 4A). It was also observed that the adsorption time for phage vB_Sau-E was ~7 min, with >80% of phage particles adsorbed to the bacterial cell wall (Figure 4B). The phage life cycle lasted ~30 min, with the assembly phase visible in the timeframe between 20 and 25 min (Figure 4C). The average number of progeny virions produced per infected host cell was estimated at ~50.

### 2.6. Phage vB_Sau-E Influence on Human Cell Lines

**Figure 5:**
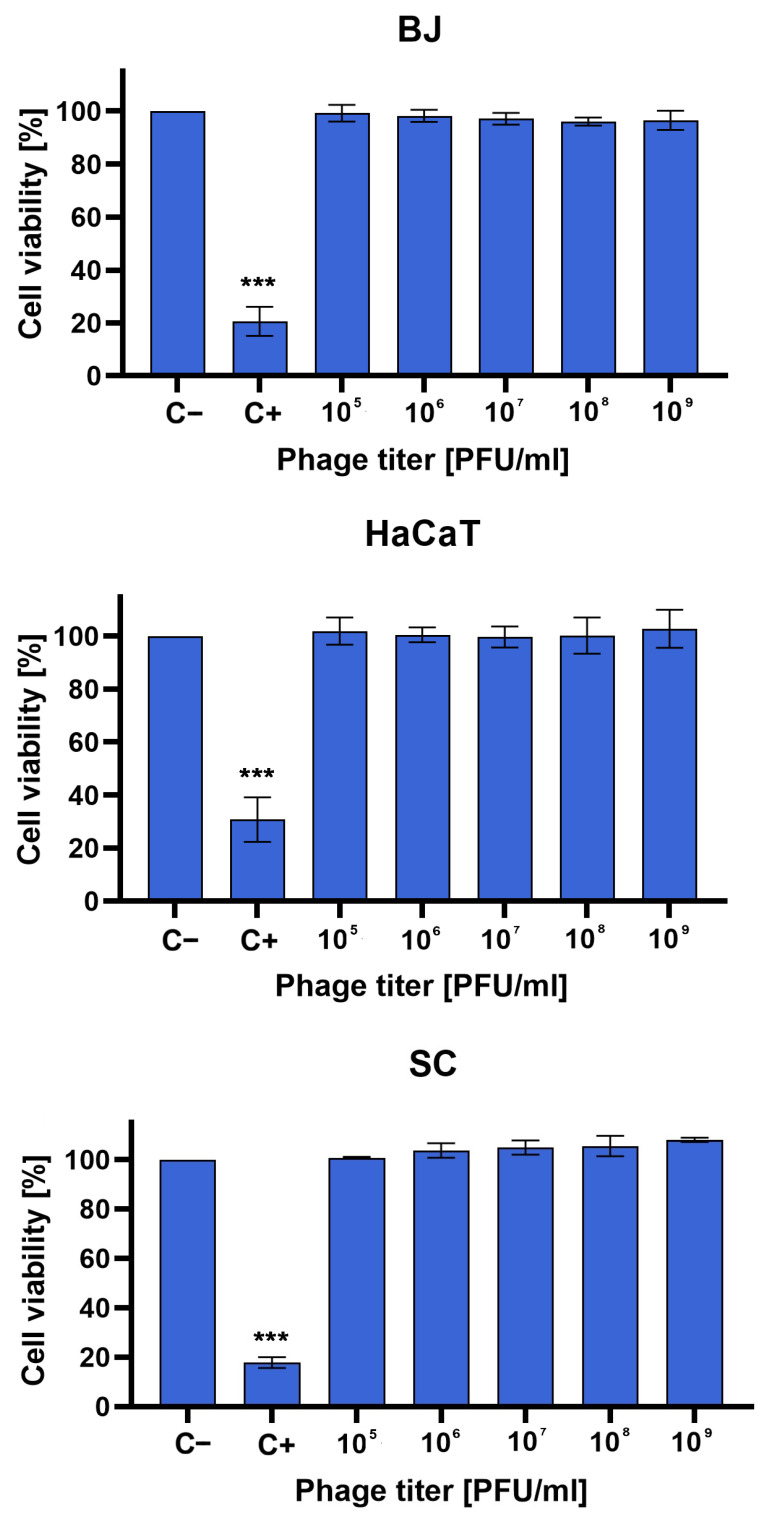
Influence of phage vB_Sau-E lysate on BJ, HaCaT, and SC cells’ viability assessed by MTT assay, in comparison to the untreated negative control (C−) and 10% DMSO-treated positive control (C+). Statistical analysis was performed using a paired t-test, with comparison of samples treated with phage to the negative control, with *** *p* < 0.001.

To assess the therapeutic potential of phage vB_Sau-E in the treatment of staphylococcal skin infections, the safety of the phage lysate to human fibroblasts (BJ), monocytes (SC), and keratinocytes (HaCaT) was analyzed. We found that a 24 h incubation of phages with the specified cell lines did not adversely affect their metabolic activity, regardless of the phage concentration used, although there was a ~4% decrease in viability of the BJ cells if two of the highest phage concentrations were used (though statistical analysis revealed this drop in viability to be insignificant) (Figure 5).

### 2.7. Lactoferrin Influence on Phage vB_Sau-E Efficacy of Plating and Survivability

To test the influence of lactoferrin on the phage efficacy, we compared phage EOP and survivability at different temperatures and pHs, with and without Lf supplementation.

**Figure 6:**
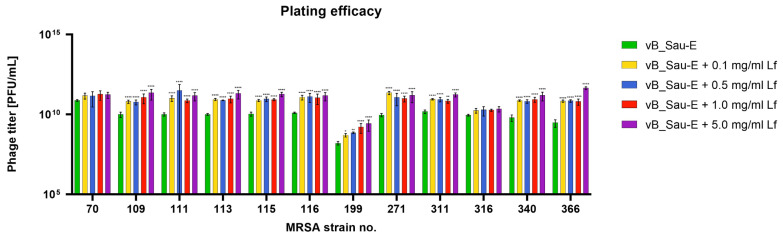
Effect of lactoferrin (0.1, 0.5, 1.0, and 5.0 mg/mL) on the efficiency of plating of phage vB_Sau-E, expressed as changes in phage titer relative to the control. Data represent mean values of triplicates ± SD. Statistical significance was determined using two-way ANOVA (* *p* < 0.05, ** *p* < 0.01, **** *p* < 0.0001).

We observed that the addition of Lf resulted in a statistically significant increase in phage titer across all but two tested strains (MRSA 70 and MRSA 316), regardless of concentration, as shown in Figure 6. The addition of Lf did not broaden the spectrum of the vB_Sau-E, as no plaques were observed on previously insensitive strains. However, on some of them, irregular clearance zones were observed.

**Figure 7:**
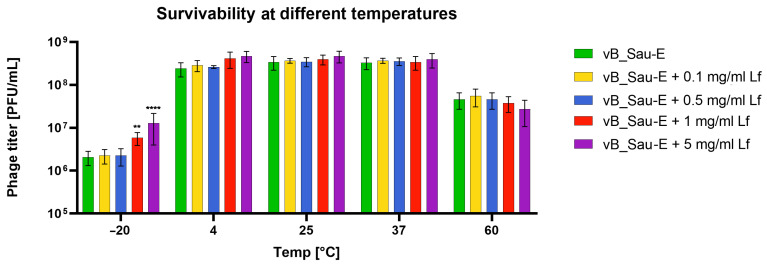
Effect of lactoferrin (0.1, 0.5, 1.0, and 5.0 mg/mL) on the thermal stability of phage vB_Sau-E, expressed as changes in phage titer relative to the control at different temperatures. Data represent mean values of triplicates ± SD. Statistical significance was determined using two-way ANOVA (** *p* < 0.01, **** *p* < 0.0001).

The experiments regarding phage survivability were performed using the MRSA strain 70, as addition of Lf resulted in no significant difference in phage EOP for this strain (Figure 6). Any changes in phage titer that were observed were most likely the result of Lf’s protective effect on the phage capsid. We have observed that while the addition of lactoferrin did not affect phage stability at positive temperatures, supplementation with 1.0 and 5.0 mg/mL of Lf resulted in improved survivability at −20 °C (Figure 7).

**Figure 8:**
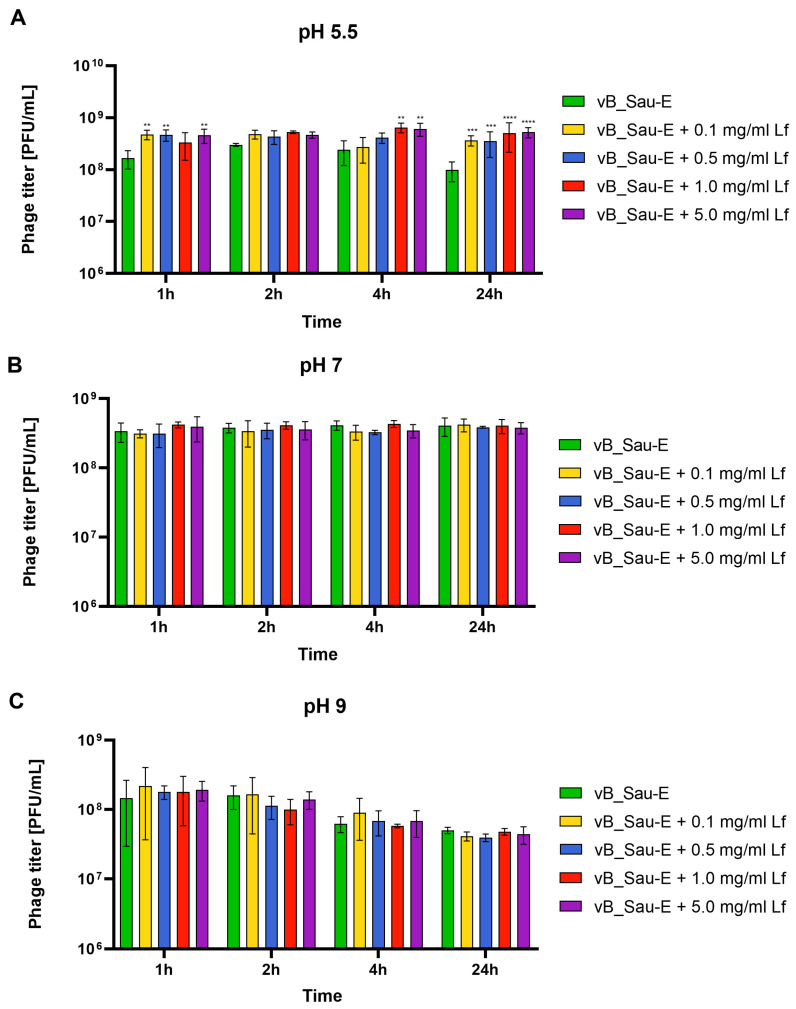
Effect of lactoferrin (0.1, 0.5, 1.0, and 5.0 mg/mL) on the survivability of phage vB_Sau-E at a pH of 5.5 (**A**), 7.0 (**B**), and 9.0 (**C**) over time. Data represent mean values of triplicates ± SD. Statistical significance was determined using two-way ANOVA (** *p* < 0.01, *** *p* < 0.001, **** *p* < 0.0001).

Additionally, at a pH of 5.5, lactoferrin supplementation consistently increased phage titers in comparison with the control in a concentration and time-dependent manner, reaching the highest levels at ≥1.0 mg/mL after prolonged incubation (Figure 8A). There were no differences in survivability observed in the case of a pH of 9 or 12 (Figure 8B,C).

### 2.8. Phage-Lactoferrin Additive Activity Assessment Using Checkerboard Assays

**Figure 9:**
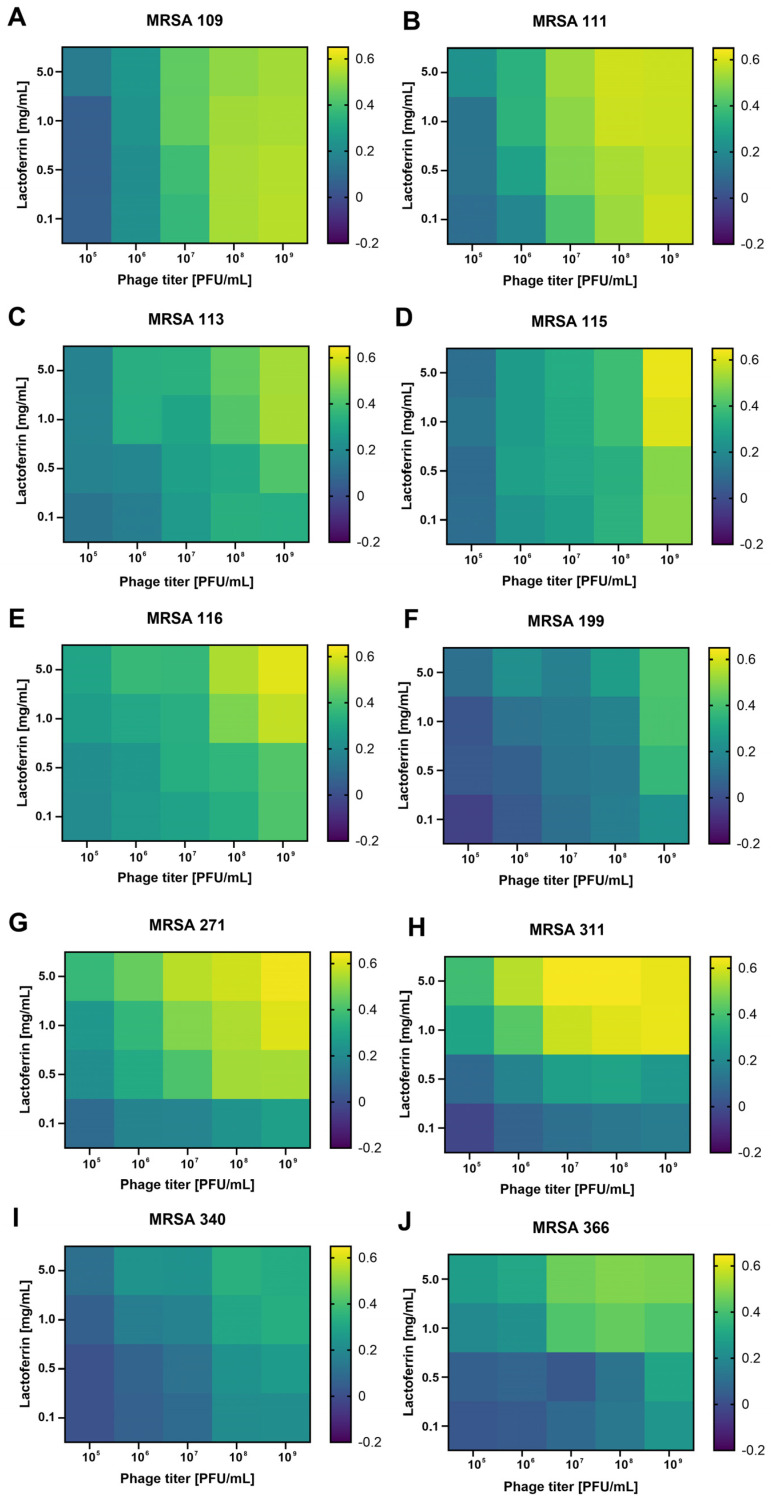
The Bliss scores (ΔE) for phage–lactoferrin synergy on different clinical MRSA isolates based on MTT biofilm viability assay: 109 (**A**), 111 (**B**), 113 (**C**), 115 (**D**), 116 (**E**), 199 (**F**), 271 (**G**), 311 (**H**), 340 (**I**). 366 (**J**). Interactions are classified as: synergistic if ΔE > 0.5, additive if 0.1 < ΔE < 0.5, no effect if −0.1 < ΔE < 0.1, or antagonistic if ΔE < −0.1.

**Figure 10:**
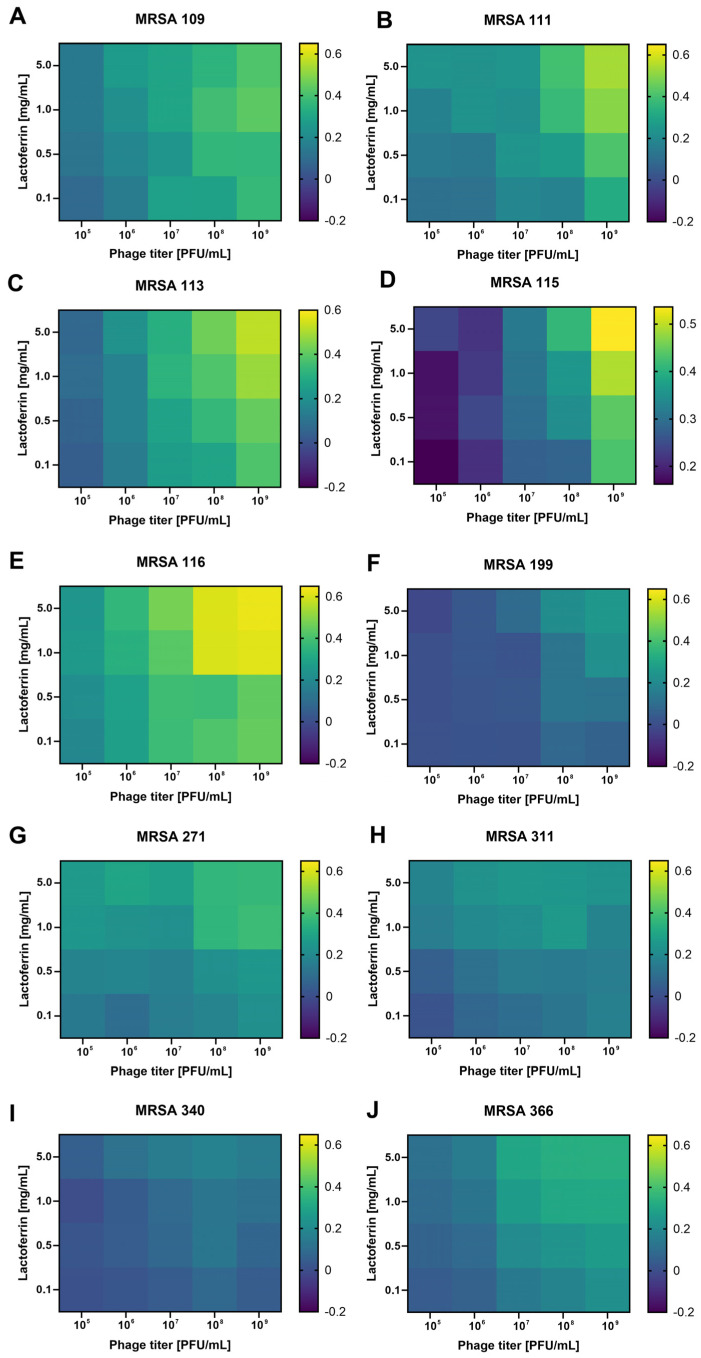
The Bliss scores (ΔE) for phage–lactoferrin synergy on different clinical MRSA isolates based on CV biofilm biomass staining assay: 109 (**A**), 111 (**B**), 113 (**C**), 115 (**D**), 116 (**E**), 199 (**F**), 271 (**G**), 311 (**H**), 340 (**I**). 366 (**J**). Interactions are classified as: synergistic if ΔE > 0.5, additive if 0.1 < ΔE < 0.5, no effect if −0.1 < ΔE < 0.1, or antagonistic if ΔE < −0.1.

Checkerboard assays were performed for 10 MRSA strains used in this study. The strains, in which an increase in EOP was not observed (70 and 316), were excluded from the assay. Across the remaining MRSA strains, increasing phage titers and lactoferrin concentrations showed both additive interactions and synergy. Bliss scores ≥ 0.5 were observed in MTT viability assay at phage titers of 10^7^–10^9^ PFU/mL combined with ≥1.0 mg/mL lactoferrin in the case of MRSA strains 109, 111, 115, 116, 271, and 311 (Figure 9A,B,D,E,G,H), while in case of the other strains the effects were mainly classified as additive (Figure 9). While analyzing the Bliss scores for the reduction in biofilm biomass assessed by crystal violet (CV) staining, Bliss scores ≥ 0.5 were observed only for MRSA strains 111, 113, 115, and 116 (Figure 10B–E).

## 3. Discussion

The increasing prevalence of antibiotic-resistant *Staphylococcus aureus* in skin and wound infections necessitates the development of alternative and/or adjunctive antimicrobial strategies. Therefore, in recent years, we have observed a rapid increase in reports describing novel phages infecting *S. aureus* [19,20,21,22]. In this study, we report the isolation and comprehensive characterization of a novel bacteriophage infecting *Staphylococcus aureus*. The phage exhibits myoviral morphology and creates small, clear plaques on bacterial lawn—typical traits of the members of the *Silviavirus* genus [23,24]. Duration of the lytic life cycle and the burst size are similar to those reported for other staphylococcal phages [25,26,27]. Phage vB_Sau-E exhibited productive infection against 12 out of 22 clinical MRSA isolates recovered from skin and soft tissue infections, indicating a relatively broad host range within this clinically relevant strain collection and consistent with other reports [28,29]. Notably, 10 of the 12 susceptible isolates were classified as good hosts, highlighting the potential therapeutic value of the studied phage as a candidate for anti-MRSA phage-based approaches.

In addition to phage characteristics, which provided primary data on phage potential as an antibacterial agent, we assessed the influence of vB_Sau-E on human skin cell lines—fibroblasts and keratinocytes—and macrophages to assess the safety of the possible treatment. We observed that while the phage presence had no negative effect on monocytes and keratinocytes, the highest phage concentrations resulted in a slight, but not statistically significant, ~4% decrease in the viability of the fibroblast cell line. A similar yet statistically significant observation was previously made in the case of myoviruses vB_SauM-A and vB_SauM-D, where treatment with 10^9^ PFU/mL of the lysate resulted in an 8–10% drop in viability [30]. However, experiments performed on murine models and case studies report that phage doses of 10^7^ PFU/mL or 10^6^ PFU/mL were usually enough to reduce the number of *S. aureus* effectively and resulted in successful treatment outcomes [9,31,32,33]. Therefore, it is possible to achieve the desired therapeutic effect without the potential negative effects of phage vB_Sau-E on cells.

Building on the observed safety and efficacy profile within therapeutically relevant phage concentrations, we next sought to evaluate if the addition of other antimicrobial compounds could enhance phage efficacy. Lactoferrin was chosen as it has well-documented antibiofilm activity [34,35]. Additionally, it has been reported that it plays a part in wound healing, and pairing Lf with other compounds would result in faster wound closure and reduction in the inflammation [36,37,38]. We have observed that the addition of lactoferrin increased the efficacy of plating of phage vB_Sau-E on most of the tested strains. Similar observations were previously made for phage vB_SauM-A [16].

An interesting observation of the present study was the increased stability of phage vB_Sau-E in acidic conditions and during storage at −20 °C in the presence of lactoferrin. This effect may be related to the strongly cationic nature of lactoferrin, which could contribute to stabilization of phage particles through electrostatic interactions [34]. Such stabilization may be relevant in infected wounds, where pH changes dynamically during infection and healing [39,40]. Additionally, lactoferrin also showed a protective effect during storage at −20 °C, indicating that its stabilizing properties are not limited to acidic environments. Freezing and thawing are known to negatively affect phage viability due to ice crystal formation, osmotic stress, and structural damage to capsid proteins [41]. The presence of lactoferrin may partially mitigate these effects through electrostatic interactions with phage particles or by creating a more protective microenvironment during freezing. Similar cryoprotective effects of Lf on phages have already been reported by Golshahi et al. while preparing the formulations for treatment of *Burkholderia cepacia* and *Pseudomonas aeruginosa* infections in cystic fibrosis patients [42]. From an application perspective, improved low-temperature stability may facilitate storage, transport, and formulation development of phage-based therapeutics intended for clinical use. However, it has to be noted that these observations remain preliminary and should be further evaluated in more complex experimental systems, including animal models.

Analysis of the checkerboard assays reveals a dose-dependent additive antibacterial effect, suggesting that lactoferrin enhances phage efficacy, potentially by altering bacterial physiology or improving phage access to cells. However, strain-dependent variability highlights the importance of intrinsic differences in phage susceptibility and biofilm properties in determining treatment efficacy.

In conclusion, our findings demonstrate that phage vB_Sau-E represents a promising addition to the growing arsenal of anti-staphylococcal phages, combining favorable lytic properties with a high safety profile in human cell models at therapeutically relevant concentrations. Importantly, the observed enhancement of phage activity in the presence of lactoferrin shows the potential of combined approaches to improve treatment outcomes, particularly in the context of biofilm-associated and antibiotic-resistant skin and wound infections. The strain-dependent variability in response highlights the need for further investigation into the underlying mechanisms of phage–Lf–host interactions. Future studies should focus on in vivo validation, optimization of dosing, and mechanistic dissection of the observed synergy.

## 4. Materials and Methods

### 4.1. Bacterial Strains

Clinical methicillin-resistant *S. aureus* isolates from skin and soft tissue infections were obtained from the Department of Medical Microbiology strain collection at the Medical University of Gdańsk [26]. All strains were cultured in Luria–Bertani broth (LB; Biomaxima, Lublin, Poland) or on LB agar plates (LA) solidified with 1.5% (*w*/*v*) agar (Biomaxima, Lublin, Poland). Soft agar used for the double-layer phage plating technique was obtained by mixing LB with 0.7% (*w*/*v*) bacteriological-grade agar (Biomaxima, Lublin, Poland).

### 4.2. Phage Isolation

Phage vB_Sau-E was isolated from urban sewage obtained from Gdansk Wastewater Treatment Plant in Poland, as described by Guzmán et al., with some modifications [43]. Briefly, approximately 10 mL of the sewage sample was combined with cultures of various *S. aureus* strains adjusted to an OD600 of 0.2. The mixtures were incubated at 37 °C with shaking at 150 rpm for 3 h or until bacterial lysis was observed. Following incubation, chloroform was added, and the samples were centrifuged at 2500× *g* for 10 min, The resulting supernatants were subsequently transferred to fresh tubes. Phage presence and activity were evaluated using the double-overlay agar plaque assay.

A single plaque obtained on the MRSA strain 70 was isolated and suspended in TM buffer (10 mM Tris-HCl, 10 mM MgSO_4_, pH of 7.2). The phage isolate was subsequently propagated as described below.

### 4.3. Phage Propagation

An overnight culture (5 mL) of the bacterial host strain grown in liquid LB medium was inoculated into 500 mL of fresh LB broth and incubated at 37 °C with shaking at 150 rpm. Upon reaching an OD600 of 0.1, the culture was infected with the phage at a multiplicity of infection (MOI) of 0.1 and further incubated at 37 °C until complete lysis was observed. For phage purification, polyethylene glycol (PEG) 8000 (BioShop, Burlington, ON, Canada) was added to the lysate to a final concentration of 10% (*w*/*v*), followed by overnight incubation with stirring at 4 °C. The precipitated material was recovered by centrifugation at 8000× *g* for 20 min at 4 °C and resuspended in TM buffer. Residual PEG8000 was removed by mixing the suspension with an equal volume of chloroform, followed by centrifugation at 8000× *g* for 15 min. This procedure was repeated until no visible PEG8000 precipitate remained. The resulting phage lysates were enumerated and stored at 4 °C [26].

### 4.4. Lactoferrin (Lf)

Lactoferrin (Lf) from bovine milk (Sigma-Aldrich, St. Louis, MO, USA) was dissolved in LB broth (Biomaxima, Gdańsk, Poland) and filtered through a 0.22 μm cellulose acetate filter (Merc, Darmstadt, Germany) to form a stock solution of 10 mg/mL. The stock solution was then stored at 4 °C for up to a week.

### 4.5. Phage Visualization Using TEM and Plaque Morphology

Transmission electron microscopy analysis of the phages was performed in the Bioimaging Laboratory, Faculty of Biology, University of Gdansk, Gdansk, Poland. Virions were negatively stained with uranyl acetate; then, micrographs were taken under a Tecnai Spirit BioTwin transmission electron microscope(Thermo Fisher Scientific, Hillsboro, OR, USA) at an accelerated voltage of 120 kV.

Plaque morphology was established by pouring the mixture of phage serial dilutions mixed with 200 µL of overnight host culture and 4 mL of soft agar on Petri dishes containing 25 mL of LA solid medium. Formed plaques were photographed and measured following 24 h of incubation at 37 °C.

### 4.6. Genome Isolation and Phylogenetic Analysis

The genome of phage vB_Sau-E was isolated, as described previously [44], using the MasterPure™ Complete DNA and RNA Purification Kit (Epicentre, Madison, WI, USA). The sample was then sequenced by GenXone Company (Poznań, Poland) using Next-Generation Sequencing (NGS) and Oxford Nanopore Technologies. Contig was assembled using the Flye algorithm and then analyzed with the PhageScope Platform [45]. The complete sequence as a .fasta file and the results of functional analysis are available in Supplementary File S1.

After the performance of BLASTn (ver. 2.17.0), 20 phages with the highest query cover and identity of over 90% were chosen for assessment of phage taxonomic relations. Furthermore, a sequence of a phage infecting *Bacillus* bacteria was used as an outer specimen within the *Herelleviridae* family. The multiple sequence alignment was created by the MAFFT algorithm [46]. The obtained alignment was then analyzed with MEGA12 software to construct a phylogenetic tree using the maximum-likelihood method and 1000 bootstrap replicates [47].

### 4.7. Host Range and EOP Determination

The host range analysis of phage vB_Sau-E was performed using the double-layer agar plate technique. Phage lysates were serially diluted in TM. Then 5 μL of each dilution was spotted onto a double-layer agar plate containing different *S. aureus* strains and left to air dry. The plates were incubated overnight at 37 °C. Following the incubation, the plaques that were formed in the bacterial lawn were counted, and the phage titer was calculated. The efficacy of plating was then established by calculating the ratio between the phage titer obtained on the tested strain and the titer on the control (host) strain [19].

To assess the effect of lactoferrin on phage EOP, lactoferrin was incorporated into the agar at final concentrations of 0.1, 0.5, 1, or 5 mg/mL, as previously described [16].

### 4.8. Temperature Stability Assay

To estimate phage stability during the thermal inactivation test, five different temperatures and incubation periods were investigated, with 37 °C used as a control value: −20 °C (24 h), 4 °C (24 h), 25 °C (24 h), 60 °C (1 h), and 95 °C (1 h). The procedure was conducted as described previously, with modifications [48]. Phage lysate was diluted with LB and incubated under conditions described above. Next, the mixture was withdrawn; serial 10-fold dilutions in TM buffer were prepared and used for plating. After overnight incubation at 37 °C, the percentage of remaining phages able to form plaques was calculated. To investigate lactoferrin’s influence on phage stability, Lf was added to the phage lysate at the final concentration mentioned above.

### 4.9. pH Stability Assay

The effect of acidic and alkaline pH on phage particles was studied using LB medium with a pH of 2, pH of 5.5, pH of 9, and pH of 12, according to a procedure described previously [48]. Phage lysate was incubated for 24 h in the given medium at 37 °C. A sample of 100 µL was collected after 1 h, 2 h, 4 h, 12 h, and 24 h of incubation, and serial 10-fold dilutions were used for plating. To determine phage stability in various pH levels, phages incubated in the medium of a pH of 7 were used as a control. Additionally, Lf was added to the phage lysate in the aforementioned concentrations to analyze its influence on phage survivability.

### 4.10. Lysis Profile

To analyze phage lytic activity, overnight bacterial cultures were diluted 1:100 in LB medium and incubated at 37 °C with shaking at 150 rpm until OD_600_ = 0.15 was reached. Phage lysate was then added at a multiplicity of infection (MOI) of 0.01, 0.1, or 1. An uninfected culture served as the control. The OD_600_ of each culture was measured at 15 min intervals over a total period of 240 min.

### 4.11. Adsorption Kinetics

The adsorption assay was conducted based on previously published methods with minor modifications [48]. Briefly, an overnight bacterial culture of the MRSA 70 strain was diluted 1:100 in LB medium and incubated at 37 °C with shaking at 150 rpm until OD_600_ = 0.1 was reached. Then, 1 mL of the culture was centrifuged at 4000× *g* for 5 s at RT. The supernatant was discarded, and the pellet was resuspended in 2 mL of fresh LB. Phage lysate was then added at a final MOI of 0.1, and the samples were incubated at 37 °C. Every minute, 100 µL aliquots were withdrawn and centrifuged at 6000× *g* for 30 s to pellet the bacterial cells. The resulting supernatants were serially diluted in TM and quantified for unadsorbed phage particles. The initial phage titer at time zero was defined as 100% non-adsorbed phages, and all subsequent measurements were compared to this value.

### 4.12. Single Life Cycle Analysis Using a One-Step Growth Experiment

The experiment was performed as described previously [26]. Briefly, an overnight bacterial culture was diluted 1:100 in fresh LB medium and incubated at 37 °C with shaking at 150 rpm until reaching an OD600 of 0.1. Subsequently, 5 mL of the culture was centrifuged at 4000× *g* for 10 min at 4 °C, and the resulting pellet was resuspended in 1 mL of LB medium. Phage particles were added at a multiplicity of infection (MOI) of 0.01 and allowed to adsorb for 5 min. To remove unadsorbed phages, the suspension was centrifuged again at 4000× *g* for 10 min at 4 °C. The pellet was then resuspended in 1 mL of fresh medium and transferred into 25 mL of LB medium (designated as time 0), followed by incubation at 37 °C. At designated time points, 100 μL samples were collected, serially diluted, and plated using the double-layer agar method. In parallel, an additional 100 μL aliquot was mixed with an equal volume of chloroform, centrifuged at 6000× *g* for 30 s to remove debris, and titrated to determine PFU/mL. Plates were incubated overnight at 37 °C. The number of infective centers was calculated by subtracting the phage titers obtained from chloroform-treated samples collected at 0, 5, and 10 min from the titers of the corresponding untreated samples. Burst size was determined as the ratio of the phage titer to the number of infective centers. Chloroform-treated samples were additionally used to estimate burst size and determine the eclipse period.

### 4.13. Cytotoxicity Assay

#### 4.13.1. Human Cell Lines

Normal human fibroblasts (BJ, ATCC^®^, Manassas, VA, USA, CRL-2522™), keratinocytes (HaCaT, Cytion, Heidelberg, Germany 300493), and monocytes (SC, ATCC^®^ CRL-3622™) were cultured at 37 °C in a humidified atmosphere containing 5% CO2. BJ, HaCaT, and SC cells were maintained in Eagle’s Minimum Essential Medium (EMEM), Dulbecco’s Modified Eagle Medium (DMEM), and Iscove’s Modified Dulbecco’s Medium (IMDM), respectively. For cytotoxicity assays, BJ and HaCaT cells were seeded in 96-well plates at a density of 1.5 × 10^4^ cells per well and incubated overnight to allow cell attachment. Subsequently, the cells were exposed for 24 h to serial dilutions of phage lysate ranging from 1 × 10^5^ to 1 × 10^9^ PFU/mL. SC cells were seeded at a density of 5 × 10^4^ cells per well and treated with the same range of phage lysate dilutions for 24 h. Cells incubated with culture medium alone served as the negative control, whereas cells treated with 10% DMSO were used as the positive control. 

#### 4.13.2. Viability Assay

Cell viability was evaluated by adding 15 µL of a 5 µg/mL MTT solution (Sigma-Aldrich, Saint Louis, MO, USA) prepared in PBS to each well, followed by incubation for an additional 2 h. For SC cells, 120 µL of isopropanol supplemented with 0.04 M HCl was then added to solubilize the formazan crystals. In the case of BJ and HaCaT cells, the culture medium was removed, and the wells were rinsed with PBS. Subsequently, 100 µL of DMSO was added to each well and incubated with shaking for 5 min. Absorbance was measured at 570 nm using a Synergy H1 plate reader.

### 4.14. Checkerboard Phage–Lactoferrin Additive Activity Assay

Biofilms were formed on 96-well polystyrene microtiter plates (Nest Biotechnology, Wuxi, China) in accordance with the previously described protocol [49]. Briefly, the overnight bacterial culture was diluted 1:100. Each well on the multiwell plate was inoculated with 200 µL of bacterial suspension and incubated for 24 h at 37 °C under static conditions to allow biofilm formation. Following incubation, the planktonic phase was carefully removed, and the wells were gently washed with 0.89% NaCl to eliminate non-adherent cells. Subsequently, preformed biofilms were exposed to phage lysate at serial dilutions ranging from 10^5^ to 10^9^ PFU/mL in combination with lactoferrin (Lf) at concentrations of 0.1, 0.5, 1.0, and 5.0 mg/mL in a checkerboard design. Plates were incubated at 37 °C, and biofilm biomass as well as metabolic activity were quantified after 24 h of treatment as described below.

#### 4.14.1. Biofilm Biomass Assessment Using Crystal Violet (CV) Staining

Following treatment, the medium was removed, and the biofilms were washed with 0.9% NaCl to eliminate planktonic cells. The remaining attached bacteria were fixed with methanol and stained with 0.1% crystal violet for 15 min. Excess stain was removed by washing the wells with distilled water, after which the plates were allowed to air dry. The bound crystal violet was subsequently solubilized using 200 µL of an ethanol–acetic acid–water solution (30:30:40), and the absorbance was measured at 595 nm using a Synergy H1 plate reader (BioTek EPOCH, BioTek Instruments, Winooski, VT, USA). [49].

#### 4.14.2. Assessment of Biofilm Metabolic Activity

The assessment of biofilm metabolic activity was performed as described previously [49]. Briefly, 20 µL of MTT solution (5 mg/mL prepared in 0.85% NaCl) was added to each well containing 180 µL of 0.85% NaCl, followed by incubation at 37 °C for 1 h. After incubation, the MTT solution was removed, and 200 µL of acidified isopropanol was added to dissolve the resulting formazan crystals. Absorbance was measured at 570 nm using a Synergy H1 plate reader. Metabolic activity was expressed as a percentage relative to the untreated control.

#### 4.14.3. Interaction Analysis

Phage–Lf interactions were evaluated using a Bliss independence model [50].

For each combination, the expected fractional inhibition ($E_{\mathrm{Bliss}}$) was calculated as

$$E_{\mathrm{Bliss}}=E_{P}+E_{L}-(E_{P}\cdot E_{L})$$

where $E_{P}$ and $E_{L}$ represent the fractional inhibition observed for phage lysate and Lf alone.

The degree of interaction was defined as

$$\Delta E=E_{\mathrm{observed}}-E_{\mathrm{Bliss}}$$

where $E_{\mathrm{observed}}$ corresponds to the experimentally measured inhibition for the combination. Interactions were then classified as synergistic if ΔE>0.5, additive if 0.1< ΔE < 0.5, no effect if −0.1 < ΔE < 0.1, or antagonistic if ΔE<−0.1, based on literature guidelines [51,52].

## Figures and Tables

**Table 1:** Host range and efficiency of plating (EOP) of phage vB_Sau-E, expressed as the percentage of phage titer relative to that obtained on the isolation (host) strain. Data represent mean values of triplicates ± SD. EOP classification: ≥100%—very good host; 99–10%—good host; 9.9–0.1%—poor host; ≤0.1%—very poor host.

MRSA Strain No.	Phage Titer (PFU/mL)	EOP (%)
**70**	4.63 (±1.11) × 10^10^	100.00 ± 0.00
**17**	0	0.00 ± 0.00
**23**	0	0.00 ± 0.00
**109**	1.01 (±0.36) × 10^10^	23.82 ± 12.30
**111**	1.03 (±0.24) × 10^10^	23.34 ± 8.27
**113**	1.01 (±0.15) × 10^10^	22.28 ± 3.26
**115**	1.09 (±0.32) × 10^10^	23.83 ± 5.62
**116**	1.27 (±0.13) × 10^10^	28.33 ± 7.25
**184**	0	0.00 ± 0.00
**196**	0	0.00 ± 0.00
**198**	0	0.00 ± 0.00
**199**	1.61 (±0.42) × 10^8^	0.35 ± 0.09
**204**	0	0.00 ± 0.00
**271**	9.33 (±2.31) × 10^9^	21.63 ± 10.33
**305**	0	0.00 ± 0.00
**311**	1.52 (±0.37) × 10^10^	34.94 ± 15.95
**316**	9.07 (±1.29) × 10^9^	19.90 ± 2.30
**340**	6.40 (±2.88) × 10^9^	15.32 ± 9.48
**343**	0	0.00 ± 0.00
**344**	0	0.00 ± 0.00
**366**	3.07 (±1.51) × 10^9^	6.93 ± 3.62
**367**	0	0.00 ± 0.00

**Table 2:** Phage vB_Sau-E stability at different temperatures and pHs.

	Phage Stability										
	Temperature [°C]						pH				
	−20	4	25	37 *	60	95	2	5.5	7 *	9	12
**Phage viability [PFU/mL]**	2.06 ×10^6^ ± 7.57 × 10^5^	2.40 × 10^8^ ± 8.72 × 10^7^	3.20 × 10^8^ ± 8.72 × 10^7^	3.13 × 10^8^ ± 8.33 × 10^7^	4.60 × 10^7^ ± 1.91 × 10^7^	0.00	0.00	1.67 × 10^8^ ± 6.43 × 10^7^	3.40 × 10^8^ ± 1.06 × 10^8^	1.53 × 10^8^ ± 6.43 × 10^7^	0.00
**Phage viability [%]**	0.63 ± 0.12	72.85 ± 13.41	103.17 ± 5.50	100.0 ± 0.00	16.08 ± 9.91	0.00	0.00	49.51 ± 11.05	100.0 ± 0.00	46.67 ± 8.25	0.00

The reference temperature and pH values are marked with asterisks (*). The reference temperature and pH were chosen based on the preferred propagation conditions of the host species.

## Data Availability

The data are available at the Bridge of Knowledge data repository: https://doi.org/10.34808/z5aq-tk81 (accessed on 10 May 2026).

## Supplementary Materials

The following supporting information can be downloaded at https://www.mdpi.com/article/10.3390/ph19060808/s1. Supplementary File S1 contains the genome sequence as a .fasta file and the results of the functional bioinformatics analysis.
